# Increase in Eyeworm Infections in Eastern Europe

**DOI:** 10.3201/eid2208.160792

**Published:** 2016-08

**Authors:** Vito Colella, Zvezdelina Kirkova, Éva Fok, Andrei D. Mihalca, Suzana Tasić-Otašević, Adnan Hodžić, Filipe Dantas-Torres, Domenico Otranto

**Affiliations:** Università Degli Studi di Bari, Bari, Italy (V. Colella, F. Dantas-Torres, D. Otranto);; Trakia University, Stara Zagora, Bulgaria (Z. Kirkova);; Szent István University, Budapest, Hungary (É. Fok);; University of Agricultural Sciences and Veterinary Medicine, Cluj Napoca, Romania (A.D. Mihalca);; University of Niš, Niš, Serbia (S. Tasić-Otašević);; University of Veterinary Medicine Vienna, Vienna, Austria (A. Hodžić);; Centro de Pesquisas Aggeu Magalhães, Recife, Brazil (F. Dantas-Torres)

**Keywords:** eyeworm, Thelazia callipaeda, nematode, parasites, thelaziosis, infections, zoonoses, eastern Europe

**To the Editor:** In the past 30 years, war in the Balkans, the fall of Communist regimes, and economic recession in Europe have undermined the economic stability of countries in eastern Europe and eventually favored occurrence of so-called neglected infections of poverty (*1*). Parasitic infections causing eye disease in persons living in areas with low socioeconomic standards might be caused by parasites not well known by healthcare providers.

A good example is *Thelazia callipaeda* (Spirurida, Thelaziidae) nematode infections in children and elderly persons living in rural and poor communities in countries in Europe and Asia (*2*). In Europe, vectors for this nematode are male *Phortica variegata* drosophilids, which feed on ocular secretions of hosts and transmit infective stage larvae to domestic and wild carnivores, lagomorphs, and humans (*3*). Possible outcomes of this infection include conjunctivitis, lacrimation, corneal ulcers, perforation, and blindness (*3*), but differentiating *T. callipaeda* infection from other ocular conditions, such as conjunctivitis-causing pathogens and allergies, can be difficult because signs and symptoms might be similar.

*T. callipaeda* was previously known as the oriental eyeworm because of its original description in countries in eastern Asia (e.g., China, Japan, and Thailand), where it has caused >1,000 cases of human infections in the past 2 decades (*2*). Since 1989, this nematode has also been detected in many countries in Europe, including Italy, France, Spain, Portugal, Switzerland, Germany, and Greece, as an agent of animal and human ocular infection (*3*). However, data on the occurrence of this parasite in countries in eastern Europe were not available until 2014.

Over the past 2 years, several autochthonous cases of ocular thelaziosis in dogs and cats (Romania, Croatia, Serbia, Bosnia and Herzegovina, Bulgaria) and foxes (Bosnia and Herzegovina) were reported (*4*–*7*) (Table). In 2016, the zoonotic potential of this parasite in those regions was further confirmed by 2 human cases of thelaziosis, one in a 36-year-old man living in Serbia (*7*) and one in an 82-year-old man living in Croatia (*8*) (Table).

**Table:** Cases of thelaziosis reported in animals and humans in eastern Europe

Country	Host	No. infected hosts	Reference
Bosnia and Herzegovina	Fox	51	(*5*)
Bosnia and Herzegovina	Dog	4	(*5*)
Bosnia and Herzegovina	Cat	1	(*5*)
Croatia	Dog	2	(*5*)
Croatia	Human	1	(*8*)
Romania	Dog	1	(*6*)
Serbia	Dog	6	(*4**,**7*)
Serbia	Cat	2	(*4*)
Serbia	Human	1	(*7*)
Hungary	Dog	1	This study
Bulgaria	Dog	9	This study

We report 10 new cases of ocular infection by *T. callipaeda* in dogs living in Bulgaria (n = 9) and Hungary (n = 1). All animals had no history of travel outside their native countries and were brought to the Department of Parasitology (Stara Zagora, Bulgaria) and to a veterinary practitioner (Pécs, Hungary) with various ocular disorders (i.e., epiphora, conjunctivitis). Nematodes detected in the conjunctival sac were collected by flushing the sac with saline solution. These nematodes were then stored in 70% ethanol and morphologically identified according to the procedure of Otranto et al. (*9*).

Molecular characterization by using PCR amplification and sequencing of a partial region of the cytochrome oxidase subunit 1 gene were performed as described (*10*). Nucleotide sequences were identical to those of *T. callipaeda* nematode haplotype-1 (GenBank accession no. AM042549), which is the only haplotype circulating in animals and humans in Europe.

Our confirmed autochthonous cases of thelaziosis in Hungary and Bulgaria have extended the geographic distribution of *T. callipaeda* nematodes from neighboring countries (e.g., Bosnia and Herzegovina, Croatia, Romania and, Greece), where occurrence of the parasite in humans and animals was already documented. Cases of human thelaziosis are reported in areas where the infection is highly prevalent in animals (*3*). Although no large-scale prevalence study has been conducted in countries in eastern Europe, 51 (27.7%) of 184 foxes in Bosnia and Herzegovina were infected with *T. callipaeda* nematodes (*5*). Isolation of *T. callipaeda* eyeworms from dogs in Bulgaria and Hungary should increases awareness of medical and veterinary communities in countries in eastern Europe for this zoonotic parasitosis. Use of a One Health approach is imperative for preventing additional eyeworm infections in persons living in eastern Europe.
